# Two-Dimensional TiO_2_ Ultraviolet Filters for Sunscreens

**DOI:** 10.1007/s40820-025-01805-1

**Published:** 2025-06-17

**Authors:** Ruoning Yang, Jiefu Chen, Xiang Li, Yaxin Zhang, Baofu Ding, Yujiangsheng Xu, Shaoqiang Luo, Shaohua Ma, Xingang Ren, Gang Liu, Ling Qiu, Hui-Ming Cheng

**Affiliations:** 1 Shenzhen Geim Graphene Center (SGC), Tsinghua Shenzhen International Graduate School (SIGS), Tsinghua University, Shenzhen, 518055 People’s Republic of China; 2 Shenzhen Key Lab of Energy Materials for Carbon Neutrality, Shenzhen Institute of Advanced Technology, Chinese Academy of Sciences, 1068 Xueyuan Road, Shenzhen, 518055 People’s Republic of China; 3 Faculty of Materials Science and Energy Engineering, Shenzhen University of Advanced Technology, 291 Louming Road, Shenzhen, 518107 People’s Republic of China; 4 Hipapa Xihe Laboratory, Run Science Park, 18 Shenzhou Road, Huangpu, Guangzhou, 510663 People’s Republic of China; 5 Shenzhen Key Laboratory of Gene and Antibody Therapy, State Key Laboratory of Chemical Oncogenomics, Shenzhen International Graduate School (SIGS), Tsinghua University, Shenzhen, 518055 People’s Republic of China; 6 Information Materials and Intelligent Sensing Laboratory of Anhui Province, Anhui University, Hefei, 230601 People’s Republic of China; 7 Shenyang National Laboratory for Materials Science, Institute of Metal Research, Chinese Academy of Sciences, 72 Wenhua Road, Shenyang, 110016 People’s Republic of China

**Keywords:** Two-dimensional, Titanium dioxide, Sunscreen, Biosafety

## Abstract

Titanium dioxide (TiO_2_) has been an important protective ingredient in mineral-based sunscreens since the 1990s. However, traditional TiO_2_ nanoparticle formulations have seen little improvement over the past decades and continue to face persistent challenges related to light transmission, biosafety, and visual appearance. Here, we report the discovery of two-dimensional (2D) TiO_2_, characterized by a micro-sized lateral dimension (~1.6 μm) and atomic-scale thickness, which fundamentally resolves these long-standing issues. The 2D structure enables exceptional light management, achieving 80% visible light transparency—rendering it nearly invisible on the skin—while maintaining UV-blocking performance comparable to unmodified rutile TiO_2_ nanoparticles. Its larger lateral size results in a two-orders-of-magnitude reduction in skin penetration (0.96 w/w%), significantly enhancing biosafety. Moreover, the unique layered architecture inherently suppresses the generation of reactive oxygen species (ROS) under sunlight exposure, reducing the ROS generation rate by 50-fold compared to traditional TiO_2_ nanoparticles. Through precise metal element modulation, we further developed the first customizable sunscreen material capable of tuning UV protection ranges and automatically matching diverse skin tones. The 2D TiO_2_ offers a potentially transformative approach to modern sunscreen formulation, combining superior UV protection, enhanced safety and a natural appearance.

**Supplementary Information:**

The online version contains supplementary material available at 10.1007/s40820-025-01805-1.

## Highlights

- Two-dimensional (2D) TiO_2_ was developed with > 99% visible light transmittance for non-whitening UV protection, outperforming conventional 0D TiO_2_ in aesthetics while matching UV-blocking efficacy.
- 2D TiO_2_ achieves ultralow skin penetration and 90% reduced reactive oxygen species generation versus 0D TiO_2_, eliminating photocatalytic toxicity and DNA damage risks.
- 2D TiO_2_ enabled tunable UVA/UVB coverage via metal doping without compromising visible light transmittance, ensuring Scientific Committee on Consumer Safety compliance and biocompatibility.
- We integrated high UV protection, natural appearance, and photoinertness into a single material, redefining aesthetic-safe sunscreen design through 2D structural innovation.

**Supplementary Information:**

The online version contains supplementary material available at 10.1007/s40820-025-01805-1.

## Graphical abstract

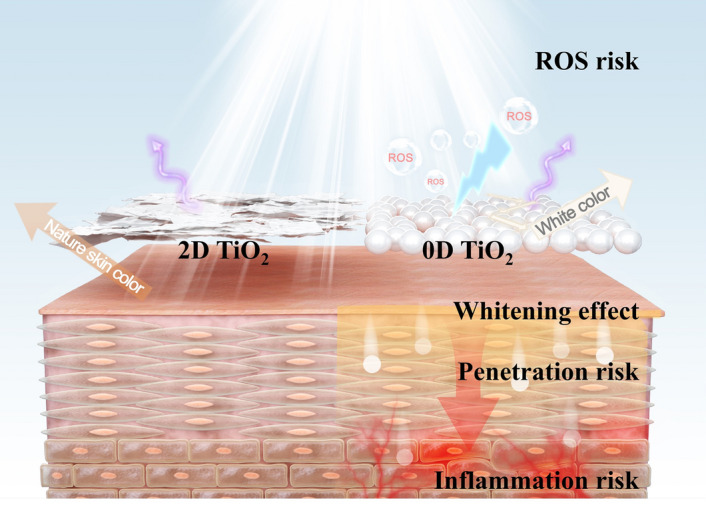

## Introduction

Skin cancer is a significant threat to human health. As reported in a World Health Organization report in 2020, there are over 1.5 million annual diagnosed cases of skin cancer worldwide, with approximately 8% of patients experiencing a fatal outcome [1]. Excessive exposure to ultraviolet (UV) radiation stands as the foremost cause, contributed to more than 90% of all skin cancer cases [2]. Human efforts to shield against UV radiation date back to ancient times. For instance, ancient Egyptians used natural jasmine oil and rice bran to shield their skin from UV damage [3]. With advances in optics and physiology during the twentieth century, systematic investigations on the effects of UV exposure on human skin were started, leading to rapid progress in the use of active ingredients for UV blocking. One of the significant milestones occurred in 1928 with the introduction of sunscreen containing salicylates and cinnamates as effective components [4]. Until 1969, avobenzone, capable of shielding against ultraviolet radiation A (UVA) in the 320–400 spectral range, was developed [5]. To date, the US Food and Drug Administration and European Commission certified sunscreens use mostly organic compounds, which are considered indispensable [6]. However, these organic UV-blocking additives are prone to degradation and loss of efficacy during photoactivation [7]. For instance, avobenzone can lose 36% of its absorption capacity after one hour of sunlight exposure [8]. Moreover, some organic additives can either penetrate the skin or cause potential skin sensitization in clinical settings [9–11].

Nowadays, inorganic titanium dioxide (TiO_2_) was gradually proposed to replace organic additives in sunscreen formulations because of its outstanding UV stability and efficient UV shielding ability [12, 13]. This significantly prolonged the UV protection time and facilitated the rise of physical sunscreen ingredients [14, 15]. Consequently, inorganic based sunscreens have gained significant market share in recent years [16]. TiO_2_ primarily exists in two crystalline phases: rutile and anatase. The anatase phase exhibits stronger phototoxicity due to its significantly distorted octahedra and is commonly utilized as a photocatalytic material [17–21], whereas the rutile phase, with higher symmetry, demonstrates reduced phototoxicity. In accordance with the Scientific Committee on Consumer Safety guidelines [22], current physical sunscreens predominantly utilize rutile-phase TiO_2_ particles in the nanometer range (> 30 nm). Current physical sunscreens mostly use spherical TiO_2_ particles with sizes in a nanometer range [23]. Although zero-dimensional (0D) TiO_2_ can absorb most of UV radiation, it also scatters a large portion of visible light due to its agglomeration, resulting in an aesthetically undesirable white cast on the skin [24, 25]. At the same time, due to the existence of a typical gap of about 300 nm between the flattened cells of stratum corneum, there is a continuously increasing concern regarding the potential penetration of 0D TiO_2_ into the human body, as most of commercially added 0D TiO_2_ has an average diameter of ~ 100 nm [26]. Pathological studies have indicated that the presence of untreated nanoscale TiO_2_ in a biological system can disrupt membrane structures [27], leading to severe issues such as DNA damage [28–30]. Moreover, nanoscale TiO_2_ particles still demonstrate unavoidable phototoxic effect, in which their photocatalytic effect can produce reactive oxygen species (ROS), leading to skin damage, even though treated by ion doping, coating, and adding antioxidants [31, 32]. Therefore, achieving a balance between a high UV-blocking efficiency, low phototoxicity and skin penetration while maintaining a natural skin appearance poses a critical challenge for the development of such sunscreens or skincare products that prioritize both aesthetics and health.

**Fig. 1:**
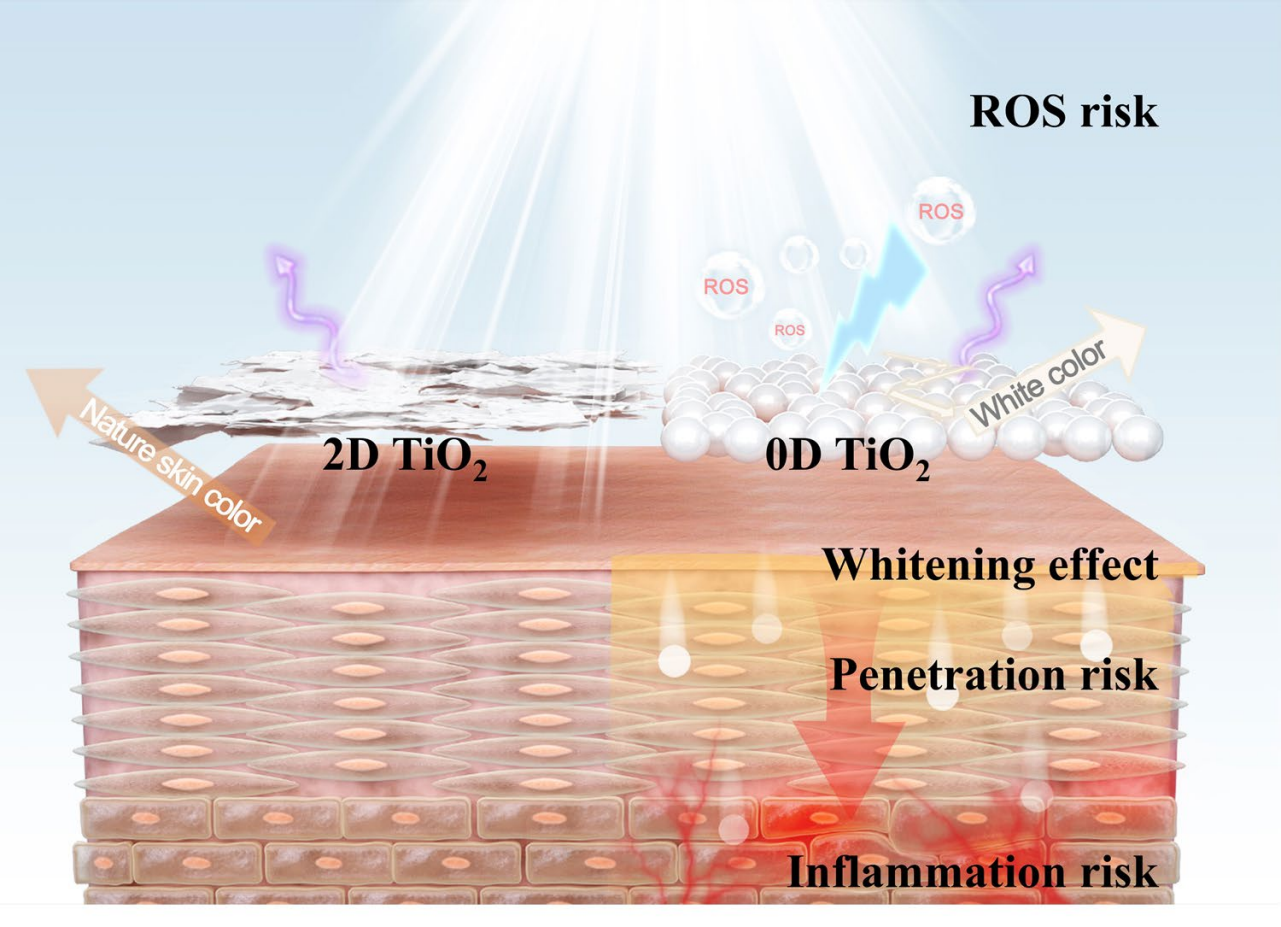
Scheme of impact of 2D TiO_2_ (left side) accompanied with natural skin color and no security risk, and 0D TiO_2_ (right side) accompanied with whitening phenomenon, free radical production and penetration on the appearance of skin surface

Here, we find that two-dimensional (2D) TiO_2_ can effectively block UV radiation while having a remarkably high visible light transmittance, extremely low skin permeability and negligible phototoxicity **(**Fig. 1**)**. In addition, the introduction of a new parameter, the Natural Appearance Factor (NAF), is discussed and applied to the 2D TiO_2_ material. NAF is a measure of how a sunscreen looks on different skin types. At the standard concentration in sunscreen products, 2D TiO_2_ has an ideal NAF value of 0.99, approaching the maximum value of 1.0, and effectively solves the problem of the white cast from conventional 0D TiO_2_. It also has both negligible phototoxicity and skin penetration, suppressing its influence on skin damage and potential health risks in vivo. More importantly, 2D TiO_2_ can also support the design of spectral coverage by doping biologically safe metals, such as iron, enabling a balanced comprehensive performance of a standard UV-blocking ratio, natural skin appearance, ultralow skin permeance, and negligible phototoxicity.

## Results and Discussion

### Optical Characteristics of 2D TiO_2_ as a UV Filter

**Fig. 2:**
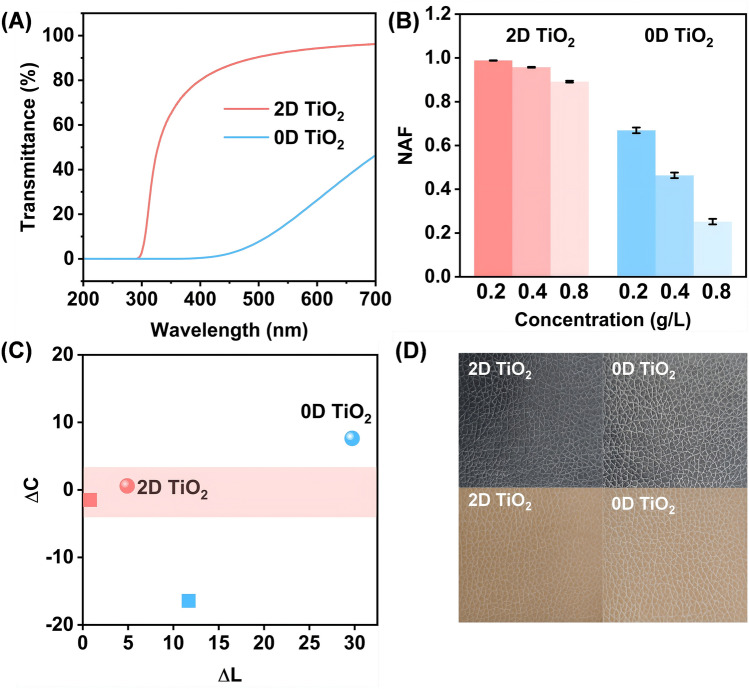
Optical characterization of 2D TiO_2_. **A** UV–visible transmittance spectra of a 0.8 g L^−1^ aqueous 2D TiO_2_ solution and 0D TiO_2_ solution. **B** NAF for 0.2, 0.4, and 0.8 g L^−1^ aqueous 2D TiO_2_ solutions and 0D TiO_2_ solutions. **C** Changing in saturation (*∆C*) and lightness (*∆L*) relative to the original leather color when coating with emulsions with a 4 w/w% solid content on synthetic leather, where the spherical shape represents the application on black leather, while the square shape represents the application on khaki leather. Smaller absolute values of *∆C* and *∆L* indicate less color alteration after emulsion coating, consistent with the requirement of a natural appearance. **D** Photographs of top views of emulsions with 4 w/w% solid content coated on synthetic leather, corresponding to **C**

2D TiO_2_ was produced by using liquid-phase ionic intercalation and exfoliation of layer-type titanate K_0.8_Li_0.27_Ti_1.73_O_4_, and in particular, a dialysis method was used to ensure the safety of the final material to the skin (see details in Method and Fig. S1) [33–35]. The 2D TiO_2_ has an average lateral size of 1.6 μm and a thickness of 1.2 nm (Fig. S2), and could be well-dispersed in water. For comparison, 0D TiO_2_ with an average diameter of ~100 nm that is commonly used in the fine chemical industry and commercial sunscreen products was selected (Figs. S3 and S4). As illustrated in Fig. 2A, 2D TiO_2_ has full ultraviolet radiation B (UVB) blocking capability in the 280–320 nm spectral range, while maintaining a transmittance of > 80% across the entire visible spectrum. In contrast, 0D TiO_2_ has a much lower overall visible light transmittance than 2D TiO_2_ due to their strong scattering. A 2D TiO_2_ dispersion of the same concentration is more transparent than that of a 0D TiO_2_ dispersion (Fig. S5). SPF is a widely accepted parameter for evaluating UV protection ability [36, 37]. Inspired by the definition of SPF, we introduce NAF, a parameter that quantitatively demonstrates the ability of a material to retain the natural appearance of a human skin (for details of its definition and calculation see discussion section S1 and Fig. S6). A NAF value close to 1 indicates a material that more accurately retains the natural appearance of the skin. The NAF values for 0.2, 0.4, and 0.8 g L^−1^ aqueous 2D TiO_2_ and 0D TiO_2_ solutions were calculated accordingly (Fig. 2B). For a given concentration, the NAF of 2D TiO_2_ is commonly much higher than that of 0D TiO_2_. As the concentration increases from 0.2 to 0.8 g L^−1^, the NAF for the aqueous 2D TiO_2_ solution decreases slightly from 0.99 to 0.89. In contrast, the NAF for the aqueous 0D TiO_2_ dispersion drops sharply from 0.67 to 0.25. This quantitatively reflects the more intense whitening with an increasing concentration of 0D TiO_2_, while 2D TiO_2_ maintain high visible light transmittance. To visually examine the difference in appearance, we prepared two emulsions containing the same concentrations of 2D and 0D TiO_2_ and then applied them to synthetic black and khaki leathers, which were used to simulate different human skin tones. By testing the difference of lightness (*∆L*) and saturation (*∆C*) of the leathers after coating with the TiO_2_ emulsions, it was clearly seen that the leather coated by 2D TiO_2_ had lower *∆L* and *∆C* values compared with the one covered with 0D TiO_2_ (Fig. 2C). And a clear visual difference could be observed in leathers coated with the two TiO_2_ concentrations, as shown in Fig. 2D.

**Fig. 3:**
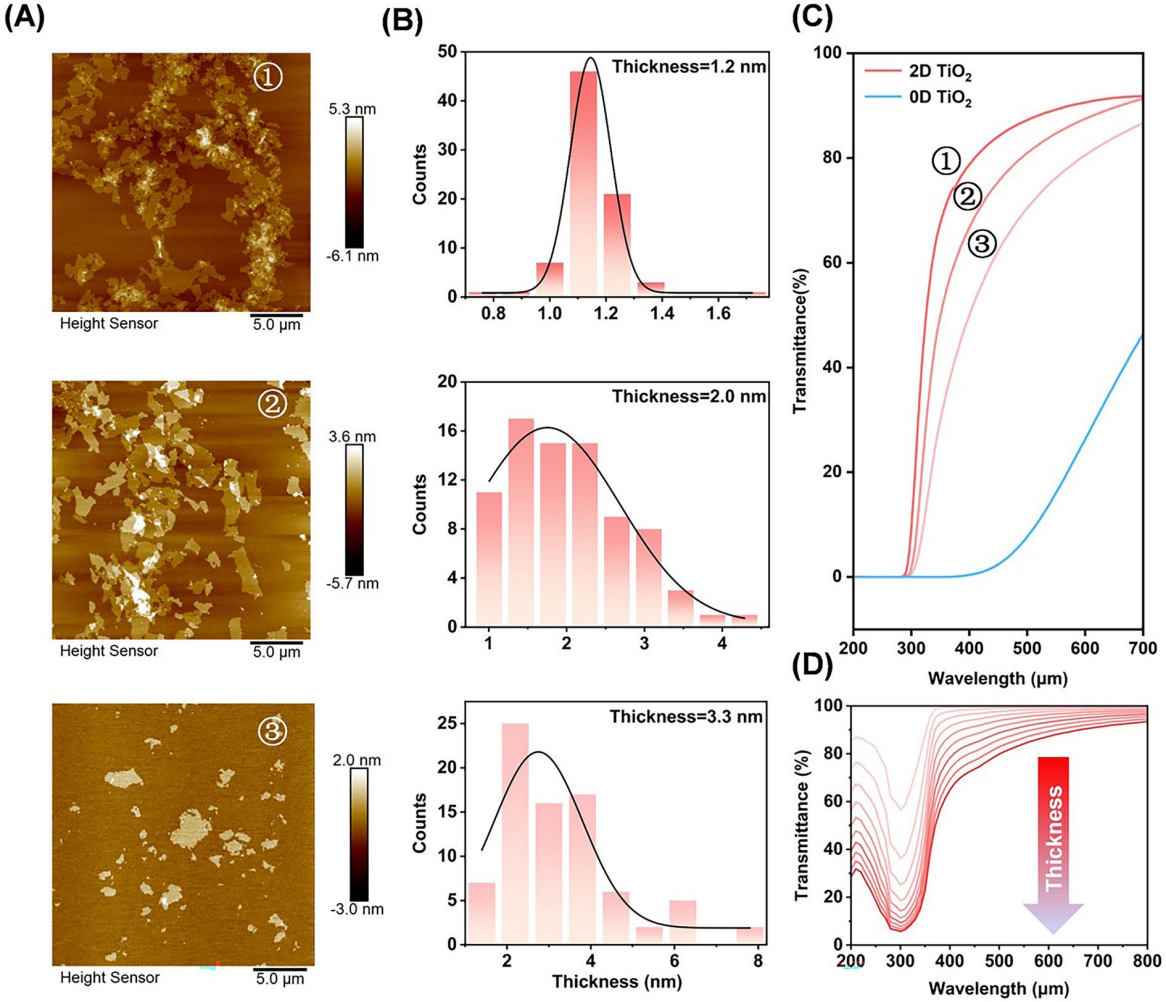
Mechanisms of the high NAF of 2D TiO_2_. **A** AFM image and **B** thickness distribution of 2D TiO_2_ under different preparation conditions, where ①, ②, and ③ are samples with different thicknesses. **C** UV–visible spectra of 2D TiO_2_ with different thicknesses and of 0D TiO_2_. **D** Simulation of the optical transmittance of 2D TiO_2_ with thicknesses of 1 to 10 nm

To study the mechanism for the high visible light transmittance of 2D TiO_2_, samples with different thicknesses were prepared by controlling the treatment time of tetrabutylammonium hydroxide. Figure 3A and B shows that a shorter treatment time results in a thicker 2D TiO_2_. The optical characteristics of 2D TiO_2_ and 0D TiO_2_ at the same concentration were determined using UV–visible spectroscopy, and it was found that reflection from 2D TiO_2_ gradually increases with increasing thickness and approaches that of 0D TiO_2_ (Fig. 3C). To understand the reason why the 2D TiO_2_ possess such high visible light transmittance, we simulated the light transmittance of 2D TiO_2_ at different thicknesses (see details in discussion section S2). As their thickness increases from 1 to 10 nm, the 2D TiO_2_ has a pronounced loss of transmittance in the range 200–400 nm (Fig. 3D), which is centered at 300 nm, the intrinsic absorption peak of TiO_2_ (Fig. S7). No significant absorption was observed in the visible light spectrum for the different thicknesses, despite a decrease in transmittance, so the loss is attributed to the increased reflectance of the sheets as their thickness grows, which substantially reduces overall transmittance. In other words, the ultra-thin thickness of 2D TiO_2_ suppresses the reflection and scattering of visible light, while maintaining UV-absorption capability.

### Low Phototoxicity of 2D TiO_2_

**Fig. 4:**
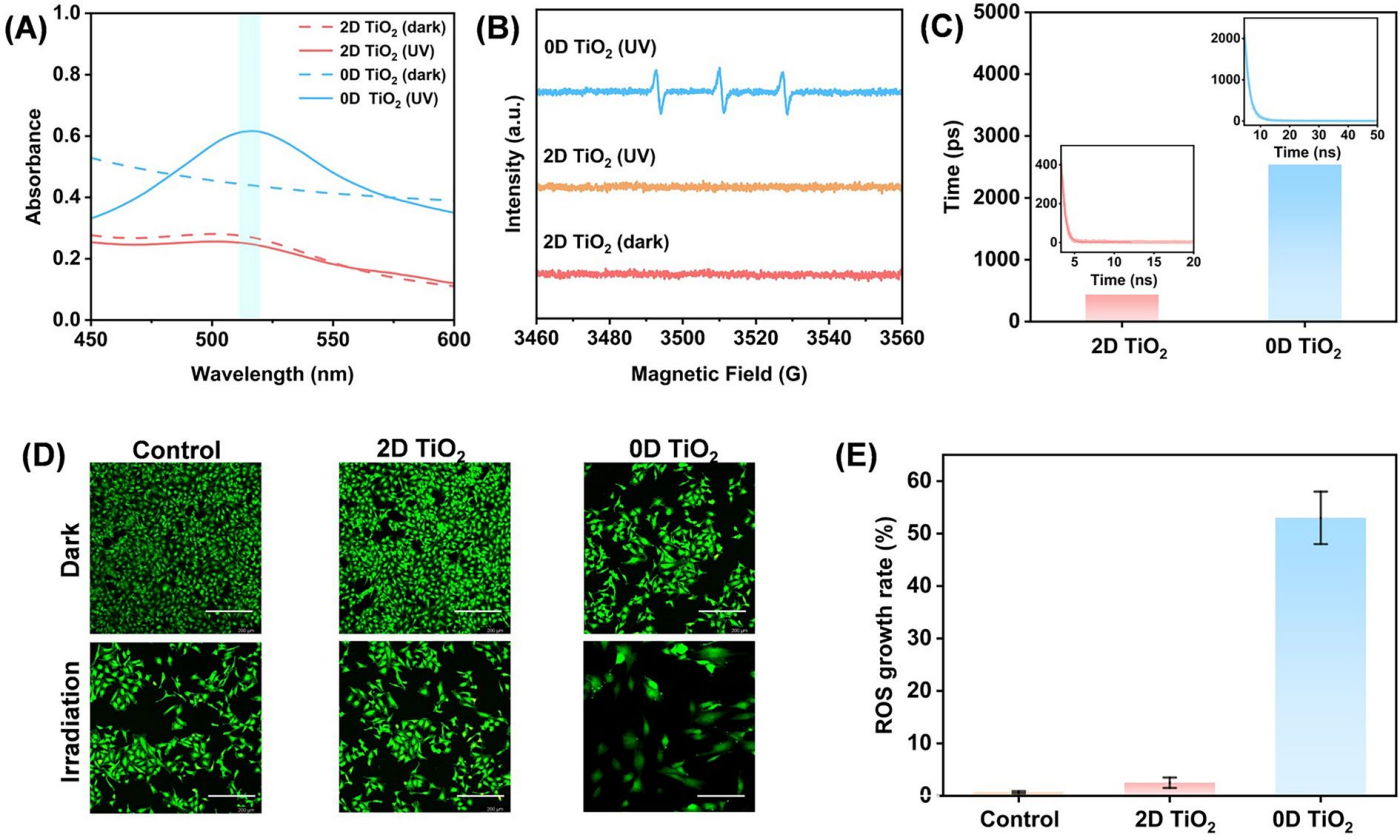
Phototoxicity of 2D TiO_2_. **A** Radical scavenging test of TiO_2_ for DPPH, showing the DPPH absorption peaks before and after UV irradiation. With equal initial concentrations of DPPH, differences in the absorption peaks under dark conditions possibly arise from the adsorption of DPPH by TiO_2_. **B** EPR analysis of reactive oxygen species generated by TiO_2_ under UV irradiation. **C** Lifetime of transient photo-generated excitons in 2D TiO_2_ and 0D TiO_2_ from photoluminescence spectra. The insets show the lifetime of excitons as a function of intensity. **D** Fluorescence images of HSF cells before and after UV irradiation. Scale bar 200 μm. **E** Effect of different types of TiO_2_ on the ROS growth rate of HSF under UV irradiation

Another health concern of the TiO_2_ additive is phototoxicity. Upon absorbing UV light, TiO_2_ generates excitons that interact with oxygen or water in the environment and consequently produce ROS. These radicals may subsequently cause phototoxic reactions on the skin to accelerate skin aging [38]. To quantitatively examine the phototoxicity of 2D TiO_2_, we conducted tests according to the Safety and Technical Standards for Cosmetics (2015). First, we used 1,1-diphenyl-2-picrylhydrazyl (DPPH) to detect free radicals generated by TiO_2_ under UV irradiation (Fig. 4A). Apart from the radical scavenging caused by the UV irradiation itself, the scavenging rate by 2D TiO_2_ (5%) was five-times less than that by 0D TiO_2_ (25%), and no detectable ROS was observed in the dispersion of 2D TiO_2_ by electron paramagnetic resonance (EPR) (Fig. 4B and Table S1).

It is important to note that the result is contrary to intuition, because the large specific surface area of 2D materials may provide more exposed active reaction sites and result in higher photocatalytic efficiency. Actually, the photocatalytic efficiency of TiO_2_ is determined by the interplay between the recombination and capture of photo-generated carriers and the interfacial charge transfer of captured carriers [39, 40]. For charge separation, holes in the valence band and electrons in the conduction band migrate to the surface of 2D TiO_2_, where they participate in the redox reactions, driving various photocatalytic processes [41]. In this regard, although 2D TiO_2_ has a larger specific surface area, it also provides more recombination centers or sites for photo-generated electrons and holes [42] This phenomenon effectively decreases the number of photo-generated free carriers, as evidenced by the much-shortened lifetime of the photo-generated excitons in 2D TiO_2_ (Fig. 4C). The phototoxicity of TiO_2_ was also assessed by cellular experiments. In co-culture with HeLa cells (Fig. S8) and human skin fibroblasts (HSF) (Fig. 4D), the cytotoxicity of both 2D and 0D TiO_2_ is low under dark conditions. When applying UV irradiation, the ROS produced by 0D TiO_2_ significantly lower the cell survival rate, while 2D TiO_2_ has a negligible influence, indicating its low phototoxicity (Fig. 4E).

### Low Skin Penetration of 2D TiO_2_

**Fig. 5:**
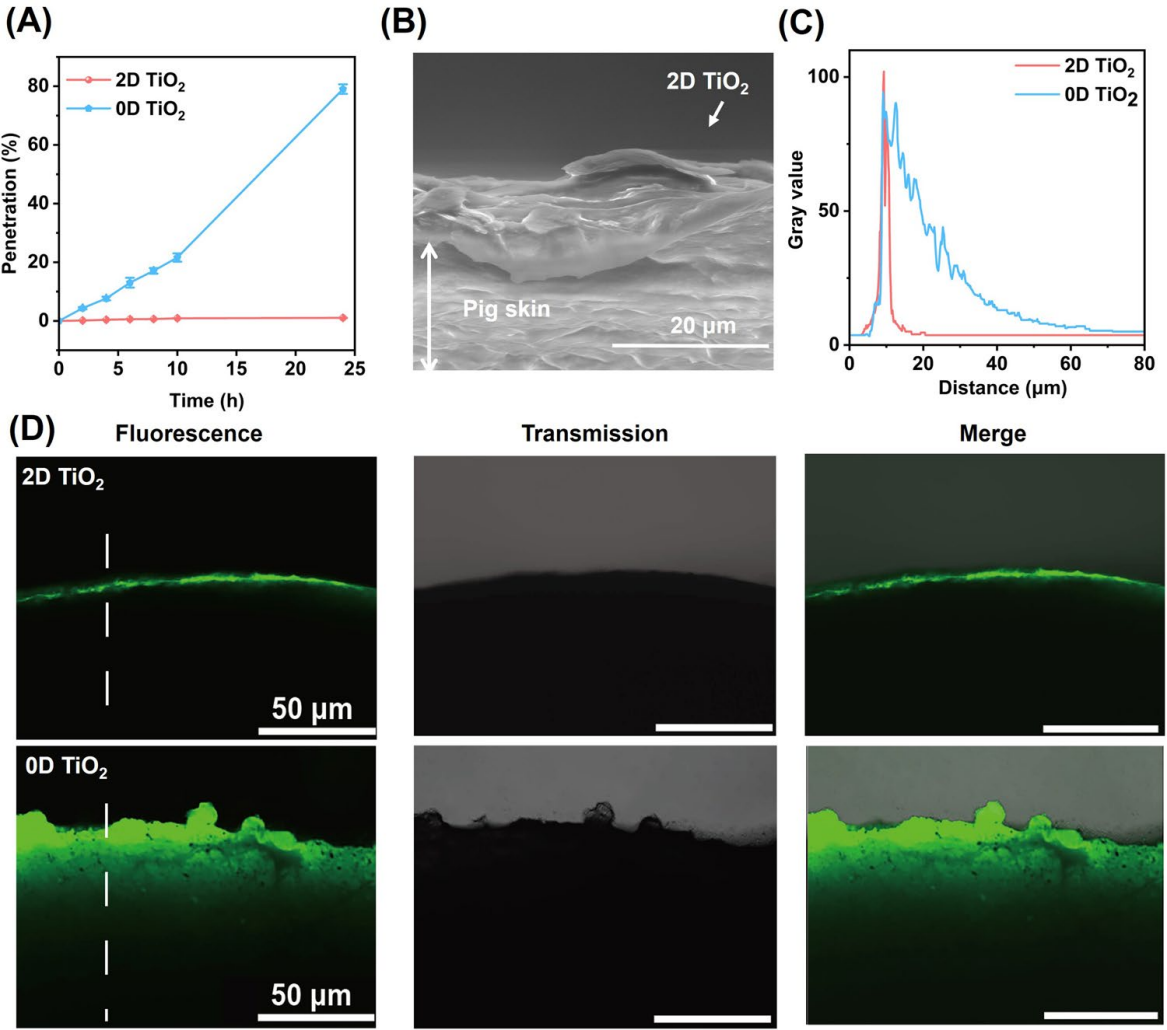
Skin penetration of 2D TiO_2_. **A** Curves depicting the mass percent of 2D and 0D TiO_2_ penetrating into a receiving pool over time in the Franz diffusion cell test, with testing time ranging from 2 to 24 h. **B** Environmental SEM image of the cross section of pig skin coated by the emulsion containing 2D TiO_2_. **C** Gray value as a function of distance, representing the fluorescence intensity and distance mapping of the dashed region corresponding to the fluorescence microscopy image in **D**. **D** Fluorescence images of labeled 2D TiO_2_ (upper ones) and 0D TiO_2_ (lower ones) on pig skin after 4 h of application

We investigated the skin permeability of 2D TiO_2_ using a Franz diffusion cell method, an established in vitro technique for assessing permeability [43] with phosphate buffered saline used to simulate the human body environment, and 3 M synthetic skin used as a skin model (Fig. S9). Samples were taken from a receiving pool, and the permeation rate was measured every two hours. After ten hours of permeation, we observed that the permeation rate of 2D TiO_2_ was approximately 0.91 w/w%, while that of 0D TiO_2_ was about 21.8 w/w% (Fig. 5A). During the subsequent 14 h (from 10 to 24 h), the permeation rate of 2D TiO_2_ remained stable at 0.96 w/w%, whereas that of 0D TiO_2_ increased up to 77.2 w/w%. These results suggest that the permeation of 2D TiO_2_ gradually decreased over time, which indicates a self-inhibitory effect of 2D TiO_2_ for preventing the diffusion of TiO_2_ into the skin.

The low permeability of 2D TiO_2_ was determined by its morphology. The sheet-like characteristic results in a structure resembling the stratum corneum when 2D TiO_2_ emulsion adhered to the surface of pig skin (Fig. 5B). However, due to a lateral size smaller than the gaps in the stratum corneum, 0D TiO_2_ can easily penetrate into deep layers of the skin, posing a potential risk of hemotoxicity and genotoxicity.

We also investigated the permeation of 2D TiO_2_ using a pig skin model, due to its close resemblance to human skin. Using a fluorescence labeling method, equal amounts of 2D and 0D TiO_2_ were applied to the surface of pig skin, and the permeation was observed by a fluorescence microscope. The dotted section in the fluorescence image was selected to generate the relationship between fluorescence intensity and penetration depth (Fig. 5C). Figure 5D illustrates the penetration depth of TiO_2_ on the pig skin surface 4 h after coating. It can be clearly seen that the 2D TiO_2_ stayed more on the pig skin surface, whereas nanoparticles penetrated it to a depth of > 20 μm.

### A Conceptual Prototype for Sunscreens

To simulate the actual use of sunscreen agents, we developed a minimalist sunscreen formula by incorporating 4 w/w% of either 2D or 0D TiO_2_ into an emulsion suitable for direct application to the skin (Fig. S10). It is known that the harmful UV spectrum affecting the skin covers UVA and UVB [44]. However, the protection provided by TiO_2_ is primarily in the UVB range due to its intrinsic electronic structure. To enhance protection, we compounded a certain amount of organic sunscreen agents to broaden the absorption wavelength to the UVA spectrum. We then investigated the effectiveness of these emulsions in protecting against UV-induced skin damage.

UV exposure is known to cause epidermal thickening and increase keratin content in the skin. Using the aforementioned emulsions as protective materials, we examined nude mouse skin tissue after UV irradiation hematoxylin–eosin (H&E) and Masson’s trichrome staining. The UV treatment lasted for 3 days, with 15 min of exposure per day. Controls included the emulsion base without UV filters and an unprotected control.

**Fig. 6:**
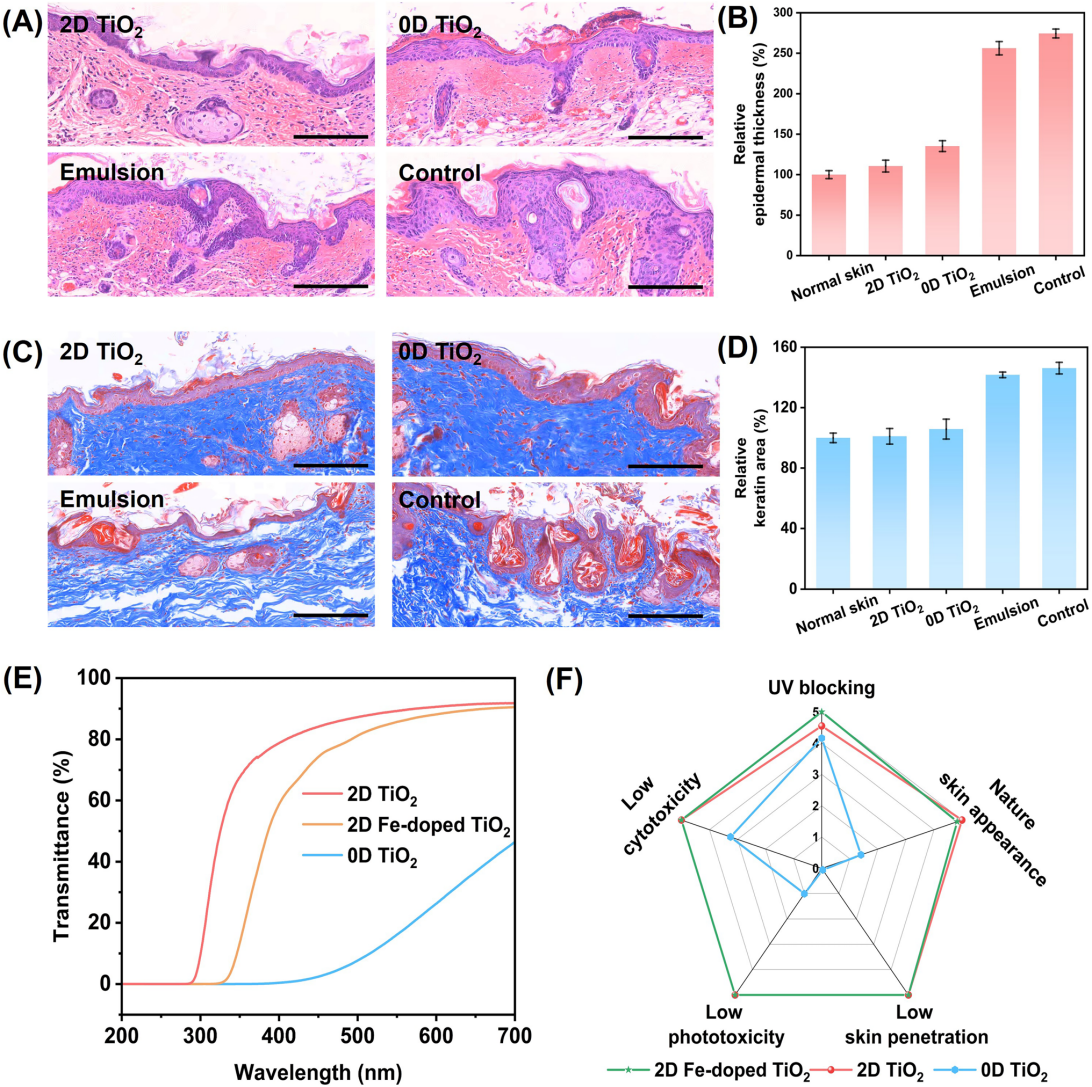
A conceptual prototype of sunscreens with 2D TiO_2_ and doped 2D TiO_2_. **A** and **B** Statistics on epidermal thickening in mouse skin and histological sections stained with H&E of sunscreens with 2D TiO_2_ and 0D TiO_2_, pure emulsion, and unprotected controls. **C** and **D** Statistics of the keratin increase in mouse skin and sections with Masson’s trichrome staining of the aforementioned sunscreens. All scale bars are 100 μm. **E** Transmittance spectra of 2D TiO_2_ doped with iron. **F** Radar graph of integrated performance of three types of TiO_2_

As shown in Fig. 6A, the epidermis of mice protected by other emulsions was twice the thickness of those protected by the emulsion with 2D TiO_2_. Quantitative analysis (Fig. 6B) shows that the epidermis of mice protected with 2D TiO_2_ remains at the same level as normal skin, while that of mice protected with 0D TiO_2_ was 120% thicker than normal skin, those of mice protected with the emulsion and unprotected mice were 256% and 275% thicker than normal skin, respectively. As shown in Figs. 6C and D, in the skin tissues of mice without the protection of TiO_2_, there were broken keratin fibers and more pores, with a significant increase in keratin content. Although the increase in keratin content in the skin tissue of mice protected with 0D TiO_2_ was not significant, severe fiber breakage and many pores were observed, affecting the original structure of the skin, likely related to the penetration of nanoparticles. In contrast, no significant increase in keratin was observed in the skin tissue of mice protected with 2D TiO_2_, indicating its effective prevention of skin keratinization.

We also compared the white-cast effect of the two proof-of-concept products. After patch testing approval (See details in data S1 and data S2), the emulsions were applied to the back of the human hand. The skin’s natural appearance in the emulsion without TiO_2_ was clearly identified. While areas coated with 0D TiO_2_ showed whitening, and those coated with 2D TiO_2_, with a previously calculated NAF of 0.89 (Fig. S11), had an appearance almost identical to the original skin. Another distinctive advantage of 2D TiO_2_ lies in its tailorable optical properties by metal doping. The incorporation of appropriate elements can broaden or red-shift the absorption peak of TiO_2_, thereby compensating for the insufficient absorption of pure TiO_2_ in the UVA range. We therefore tried to introduce iron into the TiO_2_ lattice and obtained iron-doped 2D TiO_2_ (synthesis process and morphology seen in Figs. S12-S14). The XPS spectra in Fig. S15 indicate that iron is doped in the 2D TiO_2_, making it a UV filter with a broader spectrum (Fig. 6E), which possibly supports the full use of inorganic UV-blocking additive in future sunscreens. Because of the excellent performance of the 2D material (Fig. 6F), the 2D Fe-doped TiO_2_ and its formulated emulsions maintain a high visible light transmittance (Figs. S16 and S17) and low phototoxicity (Fig. S18). In addition, compared with commercial sunscreens, the sunscreen cream with 4 w/w% 2D Fe-doped TiO_2_ has a much higher UV protection efficiency in vivo (Figs. S19 and S20).

## Conclusions

We have systematically compared the optical properties, photochemical reaction activity and penetration of 2D TiO_2_ and 0D TiO_2_. 2D TiO_2_ UV filters demonstrated many unique advantages such as desired UV damage-prevention, high visible light transmittance, extremely low phototoxicity, and minimal skin permeability. A proof-of-concept sunscreen based on a 2D TiO_2_ additive was produced with these advantages. Tailoring the UV-blocking properties of 2D TiO_2_ proved to be possible by introducing a selective dopant (Fe) to the matrix of 2D TiO_2_, while maintaining both an excellent natural skin appearance and biosafety. This work introduces a new class of UV filters that overcome the limitations of traditional sunscreen agents, such as high toxicity and whitening effects, thereby paving the way for the development of next-generation sunscreens.

## Supplementary Information

Below is the link to the electronic supplementary material.

Supplementary file1 (DOCX 6237 KB)
