# Supplementary material for: Two-Dimensional TiO2 Ultraviolet Filters for Sunscreens
Source: Nanomicro Lett. 2025 Jun 17;17:300. doi: 10.1007/s40820-025-01805-1 (PMC12173993; doi:10.1007/s40820-025-01805-1)
Supplement: Supplementary file 1 — Supplementary file1 (DOCX 6237 KB) [file 40820_2025_1805_MOESM1_ESM.docx]

Supporting Information for

**Two Dimensional TiO2 Ultra-Violet Filters for Sunscreens**

Ruoning Yang1#, Jiefu Chen1#, Xiang Li1#, Yaxin Zhang1, Baofu Ding2*, Yujiangsheng Xu3, Shaoqiang Luo3, Shaohua Ma4, Xingang Ren5, Gang Liu6, Ling Qiu1*, Hui-Ming Cheng2,6,7*

1 Shenzhen Geim Graphene Center (SGC), Tsinghua Shenzhen International Graduate School (SIGS), Tsinghua University, Shenzhen 518055, P. R. China

2 Shenzhen Key Lab of Energy Materials for Carbon Neutrality, Shenzhen Institute of Advanced Technology, Chinese Academy of Sciences, 1068 Xueyuan Road, Shenzhen 518055, P. R. China

3 Hipapa Xihe Laboratory, Run Science Park, 18 Shenzhou Road, Huangpu, Guangzhou 510663, P. R. China

4 Shenzhen Key Laboratory of Gene and Antibody Therapy, State Key Laboratory of Chemical Oncogenomics, Shenzhen International Graduate School (SIGS), Tsinghua University, Shenzhen 518055, P. R. China

5 Information Materials and Intelligent Sensing Laboratory of Anhui Province, Anhui University, Hefei 230601, P. R. China

6 Shenyang National Laboratory for Materials Science, Institute of Metal Research, Chinese Academy of Sciences, 72 Wenhua Road, Shenyang 110016, P. R. China

7 Faculty of Materials Science and Energy Engineering, Shenzhen University of Advanced Technology, 291 Louming Road, Shenzhen 518107, P. R. China

# Ruoning Yang, Jiefu Chen, and Xiang Li contributed equally to this work.

* Corresponding authors. E-mail: bf.ding@siat.ac.cn (Baofu Ding); ling.qiu@sz.tsinghua.edu.cn (Ling Qiu); cheng@imr.ac.cn (Hui-Ming Cheng)

**S1 Materials and Methods**

**S1.1 Materials**

Zero dimensional (0D) Titanium dioxide (TiO2, rutile phase), potassium carbonate, Li2CO3 were purchased from Shanghai Aladdin Biochemical Co., Ltd., Phosphate buffered saline (PBS) was purchased from Xiamen Senhope Standard Material Technology Co., Ltd., Diethylamino hydroxybenzoyl hexyl benzoate (DHHB) was purchased from Shanghai SIMP Biotechnology Co., Ltd., 1,1-diphenyl-2-picrylhydrazyl was purchased from Shanghai Macklin Biochemical Technology Co., Ltd, tetrabutylammonium hydroxide (TBAOH) was purchased from Shanghai Sigma-Aldrich Co., Ltd..

**S1.2 Method**

**Synthesis method for 2D TiO­2.** A three-stage synthesis process is used to prepare TiO­2 micro-sheets [S1]. In the initial phase, a layered compound K0.8Ti1.67Li0.13Co0.2O4 (KTLO) was prepared through high-temperature crystallization. Titanium dioxide (0.25 mol, 20 g), potassium carbonate (0.06 mol, 5.94 g), and Li2CO3 (0.010 mol, 0.67 g) from Shanghai Aladdin Biochemical Co., Ltd., were ground in an alumina crucible to a chemical stoichiometric ratio and annealed at 1100 °C for 20 hours. The second stage involved the proton exchange of Li+ and K+ with H+ to produce expanded H0.93Ti1.67Co0.2O4. One gram of the layered material was mixed with 100 mL hydrochloric acid (1 M) and stirred with a magnetic stirrer for 2 days to allow sufficient ion exchange. In the third stage, the final product of single-layer or few-layer was prepared through ion exchange between interlayer H+ and tetra-n-butylammonium ions (TBA+) [S2, S3]. The H0.93Ti1.67Co0.2O4 powder was soaked in a 10% w/v TBAOH aqueous solution and gently shaken (H+: TBA+ = 1:1).

**Synthesis method for 2D Iron-doped TiO­2.** Titanium dioxide (0.25 mol, 20 g), potassium carbonate (0.06 mol, 5.94 g), Fe2O3 (0.05 mol, 7.98 g) and Li2CO3 (0.010 mol, 0.67 g) from Shanghai Aladdin Biochemical Co., Ltd., were ground in an alumina crucible to a chemical stoichiometric ratio and annealed at 1100 °C for 20 h to obtain the precursor single crystal with iron (Fe-KTLO). Subsequently, the material was subjected to treatment with a 1 mol/L hydrochloric acid (HCl) solution for approximately three days. Excess HCl was then removed by rinsing with deionized water, resulting in the protonation of iron-doped TiO2, known as Fe-HTLO•H2O. Finally, immersion in a 10% w/v solution of TBAOH followed by agitation on a shaker facilitated the exfoliation process, yielding 2D iron-doped TiO2.

**Preparation of emulsion.** A minimalist sunscreen formula by incorporating 4 w/w% of either 2D or 0D TiO­2 into an emulsion added 1 w/w% of the organic sunscreen agent diethylamino hydroxybenzoyl hexyl benzoate (DHHB), which absorbs primarily in UVA range with a peak at 354 nm. First, 4 w/w% 2D or 2D Fe-doped or 0D TiO­2 was dissolved in water to form solution A. At the same time, shea butter was dissolved by heating to 80 degrees and then 5% surfactant and DHHB were added to form solution B. Finally, solution B was stirred for 10 minutes and added to solution A to obtain solution C. After heating to 70 degrees, solution C was homogenized at 8000 rpm for 5 min to obtain an emulsion.

**S2 Characterization**

#### SEM/AFM. The morphology and microstructure of TiO2 nanosheets were examined by a scanning electron microscope (SEM,Japan, Hitachi SU8010, 15kV) and atomic force microscope (AFM, Air tapping, Cypher ES, Oxford Instruments).

**UV-Vis spectrophotometry.** He I lamp (hv= 21.2 eV) was used as the source of the UV radiation. UV-vis diffuse spectra was obtained using a UV-2600 UV-vis spectrophotometer (Shimadzu Corporation Co., Ltd), which covers a spectral range from 300-1200 cm-1. To assess the UV-blocking efficacy of 2D TiO2, we used a dispersion concentration of 0.8 g/L in a cuvette with a path length of 1 mm to simulate a sunscreen containing 4 w/w% TiO2 adhering to the skin surface.

**Colorimeter**. The difference of lightness (*∆L*) and saturation (*∆C*) of the leathers after coating was obtained by colorimeter LS170 (Shenzhen Linshang Technology Co., Ltd). First, we prepared 0D TiO2 and 2D TiO2 emulsions with standard commercial concentrations. Then we applied the emulsions to khaki and black leathers respectively, measured and recorded the lightness and saturation before and after application using a colorimeter (each group was measured 30 times and the average value was taken), and finally obtained the *∆L* and *∆C* as an evaluation of the sample's impact on beauty.

#### Franz Diffusion Cell Test. The permeability of 0D/2D TiO2 was evaluated using a Franz diffusion cell system. For this study, the Start-M artificial skin membrane was selected to standardize the experimental conditions and enhance reproducibility. The receptor compartment was filled with deionized water and maintained at 37 °C using a circulating water bath. The donor compartment was loaded with 2 mL aliquot of a titanium oxide nanosheet suspension at a concentration of 1 g/L and sealed. At predetermined time intervals (5 h, 10 h, 15 h and 20 h), aliquots of 5ml were withdrawn from the receptor compartment and immediately replaced with fresh receptor fluid. The withdrawn samples were analyzed using UV-Vis spectrophotometry to determine the concentration of 0D/2D TiO2.

#### Confocal Laser Scanning Microscopy (CLSM). Fluorescence distribution within the TiO2 was visualized using a confocal laser scanning microscope (CLSM, Japan, Olympus Corporation). Samples were prepared by staining with FITC. Excitation was performed using a 488 nm wavelength, and emission was detected within 500-550 nm. The z-stack images were captured with a 40x oil immersion lens and a step size of 0.5 µm. Image processing and analysis were conducted using Image J software. In detail, we grafted FITC onto 0D and 2D TiO₂ separately using a silane coupling agent, formulated each into emulsions, and applied them onto two porcine skin samples. After sectioning the skin with a scalpel, cross-sectional penetration was observed by fluorescence microscopy. For quantitative analysis, the fluorescence images were converted to grayscale via Image J, and the corresponding grayscale values at different depths were extracted to quantify the penetration depth of the UV-blocking components.

**Environmental scanning electron microscopy**. The morphology of TiO2 emulsion applied on pig skin was characterized by environmental scanning electron microscopy (ESEM, QUANTA2200, USA). First, we prepared 2D TiO2 emulsions with standard commercial concentrations. Then we evenly applied the emulsions to porcine skin (Bama miniature pig skin, 1-month-old piglets). After air-drying at room temperature for several minutes (to prevent excessive moisture from affecting imaging resolution), ESEM analysis was performed.

#### Electron Paramagnetic Resonance (EPR). The presence and dynamic behavior of unpaired electrons in 0D/2D TiO₂ systems were investigated through EPR spectroscopy. Specifically, the sample (1 mg) was dispersed in 1 mL of aqueous or methanolic solution, followed by the addition of 10 μL of 2,2,6,6-Tetramethylpiperidine as singlet oxygen probe. After incubation in the dark or under light irradiation, aliquots were collected using a quartz capillary (50 μL capacity). The mixture was subjected to visible light irradiation (λ ≥ 420 nm) for 10 minutes using a 300 W xenon lamp equipped with a 420 nm cutoff filter. EPR measurements were immediately conducted post-illumination at room temperature with a Bruker EMX PLUS-6/1 spectrometer operating at X-band microwave frequency (9.5 GHz), employing optimized parameters: microwave power 10 mW, modulation amplitude 1 G, and 10 consecutive scans (10 s per scan) to enhance signal-to-noise ratio (SNR). Three negative control groups were systematically established: (1) dark control (no illumination), (2) superoxide anion quenching group (with 50 U/mL superoxide dismutase, SOD), and (3) singlet oxygen quenching group (with 5 mM sodium azide, NaN₃), to eliminate cross-interference from O₂⁻• and ¹O₂ species.

#### Photoluminescence (PL) Spectroscopy. Photoluminescence (PL) spectra of TiO2 were recorded using a PL spectrometer (Hitachi F4600) equipped with a charge-coupled device (CCD). The excitation was carried out using a xenon lamp, laser at a wavelength of 325 nm.

**Human Patch Test.** To assess the skin irritation potential of TiO2, a human patch test was conducted according to the guidelines outlined in the 2015 Edition of the Cosmetics Regulation. This study was approved by the E, and all participants provided written informed consent prior to the study.

Participants. A total of 6 healthy volunteers (both male and female) aged between 18-35 years were recruited for this study. Participants were screened to ensure they had no history of dermatological diseases, allergies, or hypersensitivity reactions. Individuals who were pregnant, breastfeeding, or using any skin treatment medications were excluded from the study.

Patch Test Procedure. The patch test was performed using occlusive patches that contained 0.5 ml 25% 2D TiO2 emulsion. The patches were applied to the upper back area of each volunteer, as this site is less prone to irritation due to minimal friction and exposure to external factors. Each test substance was applied on a separate patch to avoid cross-contamination. The patches were left in place for 24 hours under occlusive conditions. After the exposure period, the patches were removed, and the skin reactions were assessed at 24 hours post-removal. The evaluation was conducted by a trained dermatologist under standardized lighting conditions.

Scoring and Evaluation: Skin reactions were scored using a standard grading scale based on erythema, edema, and other visible signs of irritation as follows:

0: No reaction

1: Mild erythema (faint, but definite redness)

2: Moderate erythema (well-defined redness)

3: Severe erythema (extreme redness)

4: Very severe erythema (deep redness with possible blistering or swelling)

Any other reactions such as dryness, peeling, or itching were also recorded. The results were documented and statistically analyzed to determine the irritation potential of each test substance.

Safety Precautions: All procedures were conducted under medical supervision to ensure participant safety. Emergency medical facilities and personnel were available in case of adverse reactions. Participants were instructed to report any discomfort or unexpected reactions immediately, and any participant who experienced severe reactions was withdrawn from the study and provided with appropriate medical care.

Data Analysis: The data obtained from the patch test were analyzed using SPSS. Descriptive statistics were calculated for each test sample, and the incidence of reactions was compared across different groups using Chi-square test. A p-value of less than 0.05 was considered statistically significant.

**Hela cell.** Pre-treatment (Cell Revival). A water bath was preheated to 37°C for cell thawing. Cryopreserved Hela cells stored at -80°C were rapidly thawed by gentle agitation in the water bath for 2 min. After complete thawing, the cell suspension was transferred to a 15 mL sterile centrifuge tube containing 7 mL of pre-warmed culture medium (DMEM supplemented with 10% FBS) and centrifuged at 1000 rpm (300 × g) for 5 min at room temperature. The supernatant was discarded, and the cell pellet was resuspended in 5 mL of fresh medium (10% FBS) before transfer to a 25 cm² cell culture flask. Cells were maintained in a humidified incubator (5% CO₂, 37°C) for 8 h to ensure adherence, followed by replacement with 5 mL of fresh medium (10% FBS). Subsequent experiments were initiated upon reaching 80–90% confluence.

For cytotoxicity evaluation via the MTT assay, Hela cells in logarithmic growth phase were trypsinized to generate a single-cell suspension in complete medium. The cell density was adjusted to 1 × 10⁵ cells/mL, and 100 μL aliquots were dispensed into each well of a 96-well plate. After 12 h of incubation (5% CO₂, 37°C), 50 μL of test sample solutions (0.1–100 μg/mL in triplicate wells) were added to designated wells. Following 24 h of treatment, the medium was aspirated, and cells were washed twice with PBS (pH 7.4). Subsequently, 100 μL of MTT working solution (0.5 mg/mL in PBS) was added to each well, and plates were incubated in the dark (37°C, 4 h). The MTT solution was replaced with 150 μL of DMSO to solubilize formazan crystals, and plates were agitated (400 rpm, 10 min) in the dark. Absorbance was measured at 570 nm using a microplate reader. Cell viability (%) was calculated as (OD Experimental / OD Control) × 100%, where untreated cells served as the control (100% viability).

**Animals and treatment.** Nude male mice (about 6-8 weeks old and 25-30 g) were purchased from the Beijing Vital River Laboratory Animal Technology Co., Ltd. (Beijing, China). They were housed in plastic cages with a stainless steel mesh lid in a ventilated room. The room was maintained at 20 ± 2 °C and 60 ± 10% relative humidity, with a 12 h light–dark cycle. Mice were fed on water and sterilized food. Prior to treatment, mice were kept fasted overnight.

Three mice were divided into three groups. Six areas were taken from the backs of each group of mice, with one area remaining unaffected by ultraviolet radiation and labeled as the normal skin group. The other five areas, corresponding to the control group, pure cream group, 2D Fe doped TiO2 group, 2D TiO2 group, and TiO2 nanoparticle group, respectively, were exposed to ultraviolet radiation (216 J cm2) for three days, with each exposure lasting 20 minutes. Mice in each group were killed on day 4. The epidermal tissue of the six marked areas were collected. Fractions of tissues were kept in 10% (v/v) formalin for immediate histopathological examination.

**Histopathological Examination.** Histological observations were performed according to the standard laboratory procedures. Mice at the end of day 4 were dissected for histology. A small piece of epidermis fixed in 10% (v/v) formalin was embedded in a paraffin block, sliced into 5 μm thicknesses and then placed onto glass slides. The section was stained with hematoxylin–eosin (HE) and masson staining, respectively, and then examined by light microscopy.

**Flow CytoMetry test.** HSF (human skin fibroblasts) cells were cultured in a 5% CO2, 37 ℃ constant temperature incubator. There are a total of 8 samples, all of which are solid. Weigh an appropriate amount of sample, sterilize with high temperature and high pressure steam (121 ℃, 20 min), cool to room temperature, add complete culture medium at a concentration of 0.2 mg/mL, sonicate all samples except A1 and A2 for 10 minutes, and store for future use. The experiment was divided into Blank, ROS-UP (positive control), light group, and non light group. The light group and non light group were evenly divided into A1-A4 and B1-B4 groups. Blank, ROS-UP (positive control), and Control groups were added with 2 mL of complete culture medium; Add 2 mL/well of corresponding sample working solution to groups A1~A4 and B1~B4. The light group was irradiated with UVA (1.5 mW/cm2) and UVB (0.011 mW/cm2) for 15 minutes each, and then cultured for 1 hour. Take HSF cells in logarithmic growth phase, perform cell counting, adjust cell concentration, and inoculate them into a 6-well culture plate at 3×105/well. Incubate overnight in a constant temperature incubator with 5% CO2 at 37 ℃ to allow the cells to adhere to the wall. Process according to the above grouping. At the end of cultivation, the ROS-UP (positive control) group discarded the culture medium, washed twice with PBS, added 2 mL of reactive oxygen species positive control working solution, and incubated for 30 minutes. After incubation, all groups were discarded from the culture medium, washed once with PBS, and digested with trypsin to collect cells. After washing once with 1 mL/tube of PBS, 500 μL of DCFH-DA working solution (10 μM) was added and incubated for 20 min; Blank group without DCFH-DA staining. After staining, wash once with 1 mL/tube of PBS and immediately test on the machine. Flow cytometry GRN-B channel was used to detect ROS fluorescence intensity.

**Discussion section S1**

The definition of the Sun Protection Factor (SPF) is

|  | $SPF=\frac{\int_{280nm}^{400nm} E_{\lambda}S_{\lambda}d\lambda}{\int_{280nm}^{400nm} E_{\lambda}S_{\lambda}T_{\lambda}d\lambda}$ | (S1) |
| --- | --- | --- |

where $E_{\lambda}$ is erythema effect factor, $S_{\lambda}$ is solar spectrum and $T_{\lambda}$ is transmittance. The erythema effect factor reflects the human response to different wavelengths, combining in vivo and in vitro tests.

The Natural Appearance Factor is defined as:

|  | $NAF=\frac{\int_{400nm}^{700nm} R_{\lambda}S_{\lambda}T_{\lambda}d\lambda}{\int_{400nm}^{700nm} R_{\lambda}S_{\lambda}d\lambda}$ | (S2) |
| --- | --- | --- |

Where $R_{\lambda}$ is the parameter of the human body's ability to reflect light at different wavelengths, with Asian, African, light and deep Caucasian as reference, and the final results are average.

**Discussion section S2**

The optical properties of micro-sheets can be obtained by solving Maxwell's equations. Here, the rigorous coupled-wave analysis (RCWA) method, which is based on Floquet’s mode theory [S4, S5], is employed to provide an efficient tool for investigating optical properties for single-layer and multi-layer oxidized micro-sheets.

**Scheme S1** gives the schematic illustration of multi-layered structure, in which the top region I and bottom region III are homogenous media. Here, region II can be one layer or more than one layer. In general, region II is typiclly considered as one dimensional (1D) grating structure with a periodicity of Λ*x*, when we set *f* =1, the region II will be degraded as homogenous medium.

**Scheme S1** A schematic of grating structures. *x* is the period in *x*-direction, *f* *x* is width of grating strip, *εgr*and *εrd*are permittivities of groove and ridge region of grating respectively, *θ* is incident angle of light.

Taking transverse magnetic (TM) polarization wave (electric field is along *x*-direction) as example, incident magnetic field is written as:

$H_{inc,y}=\exp[-jk_{0}n_{\text{I}}(\sin\theta x+\cos\theta z)]$ (S3)

The normalized fields in region I (*z<0*) and III (*z>d*) are expressed as:

$H_{\mathrm{I},y}=H_{\mathrm{inc},y}+\sum_{i} r_{i}\exp[-j(k_{xi}x-k_{\text{I},zi}z)]$ (S4)

$H_{\mathrm{III},y}=\sum_{i} t_{i}\exp\{-j[k_{xi}x+k_{\text{III},zi}(z-d)]\}$ (S5)

where $k_{\text{I},zi},k_{\text{III},zi}$ and $k_{xi}$are wave numbers and can be determined by dispersion relation and Floquet theorem. *ri* (*ti*) (*i* is the integer) is normalized magnetic field amplitudes of the *i-*th reflected (transmitted) wave in region I (III) which are unknowns.In grating region II (*0<z<d*), the periodic permittivity is expanded in Fourier series as $\varepsilon_{rd}(x)=\sum_{h} \tilde{\varepsilon}_{h}\exp(j2\pi/\Lambda_{x})$ where $\tilde{\varepsilon}_{h}$ is the *h-*th Fourier component of permittivity in grating region. The tangential magnetic (*y*-component) and electric fields (*x*-component) can be expanded by Fourier series in terms of space harmonics fields:

$H_{\text{II},y}=\sum_{i=-N}^{N} U_{yi}(z)\exp(-jk_{xi}x)$ (S6)

$E_{\text{II},x}=j\eta_{0}\sum_{i=-N}^{N} V_{xi}(z)\exp(-jk_{xi}x)$ (S7)

where *Uyi*(*z*) and *Vxi*(*z*) are the normalized amplitudes of the *i*-th space harmonic fields and *η0* is wave impedance in free space. Substituting **Eqs. S3-S7** into Maxwell’s equations, a second order matrix differential equation is obtained:

$\frac{\partial^{2}}{\partial\tilde{z}^{2}}\mathbf{U}_{y}=\mathbf{\varepsilon\Omega U}_{y}$ (S8)

where $\tilde{z}=k_{0}z$ and $\mathbf{\Omega}=\mathbf{K}_{x}\mathbf{\varepsilon}^{-1}\mathbf{K}_{x}-\mathbf{I}$ is expressed in terms of wave vectors and Fourier components of permittivity, **K***x* is a diagonal matrix of *kxi* and **ε**-1is the matrix formed by $\tilde{\varepsilon}_{h}$, **I** is unit matrix. With calculating the eigenvalues and eigenvectors of **Ω**, matching the boundary condition at *z*=0 and *z*=*d*, unknowns of *ri* and *ti* can be analytically determined. The reflection and transmission of grating structures are respectively determined by:

$R=\sum_{i} r_{i}r_{i}^{*}\mathrm{Re}[\frac{k_{I,zi}}{k_{0}n_{I}\cos\theta}]$ (S9)

$T=\sum_{i} t_{i}t_{i}^{*}\mathrm{Re}[\frac{k_{\text{II},zi}}{n_{\text{II}}^{2}}\frac{n_{\text{I}}}{k_{0}\cos\theta}]$ (S10)

Therefore, once we get the parameters include the wavelength-dependent permittivities of the micro-sheets, as well as the width and thickness of the geometric structure. The reflection and transmission of multi-layered structure can be computed and the absorption coefficient can be derived by subtracting the reflection and transmission coefficients from the normalized incident wave power.

**Supplementary Figures**


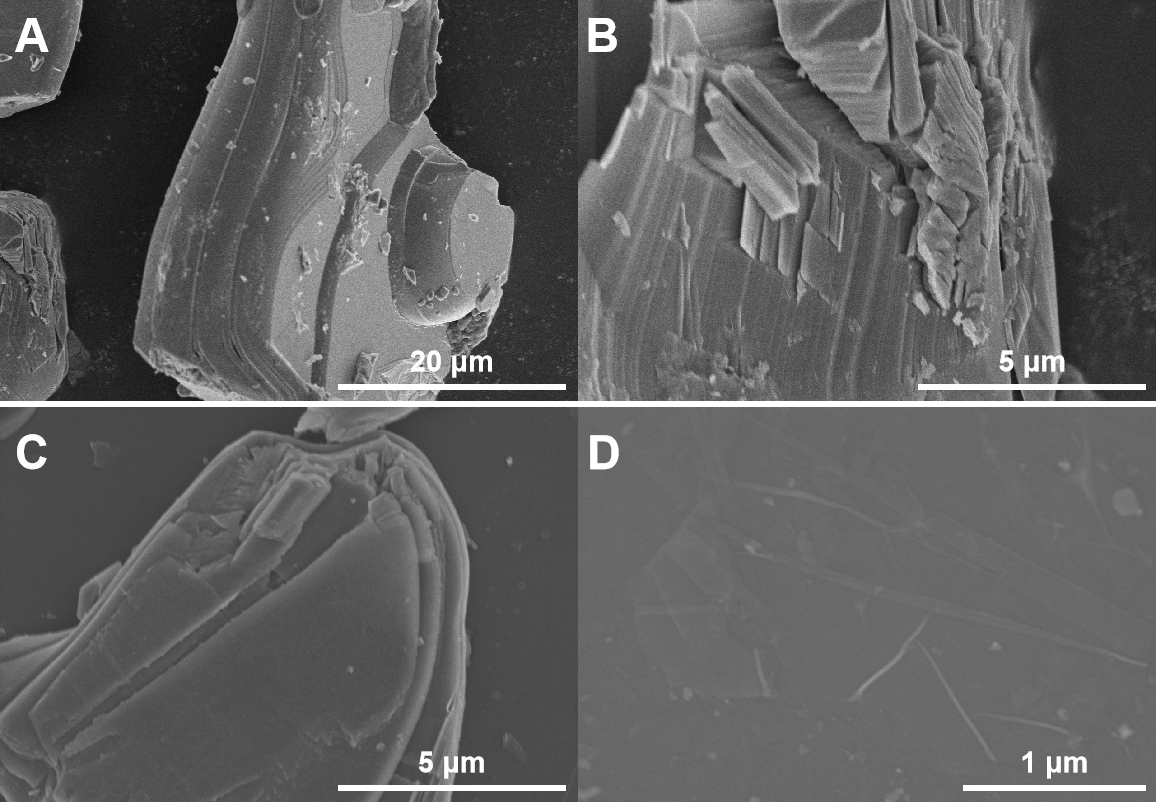


**Fig. S1** Synthesis process of 2D TiO2. (**A**) Pre-sintered KTLO. (**B**) Precursor after hydrogen ion intercalation. (**C**) Precursor with water molecule intercalation. (**D**) 2D TiO2


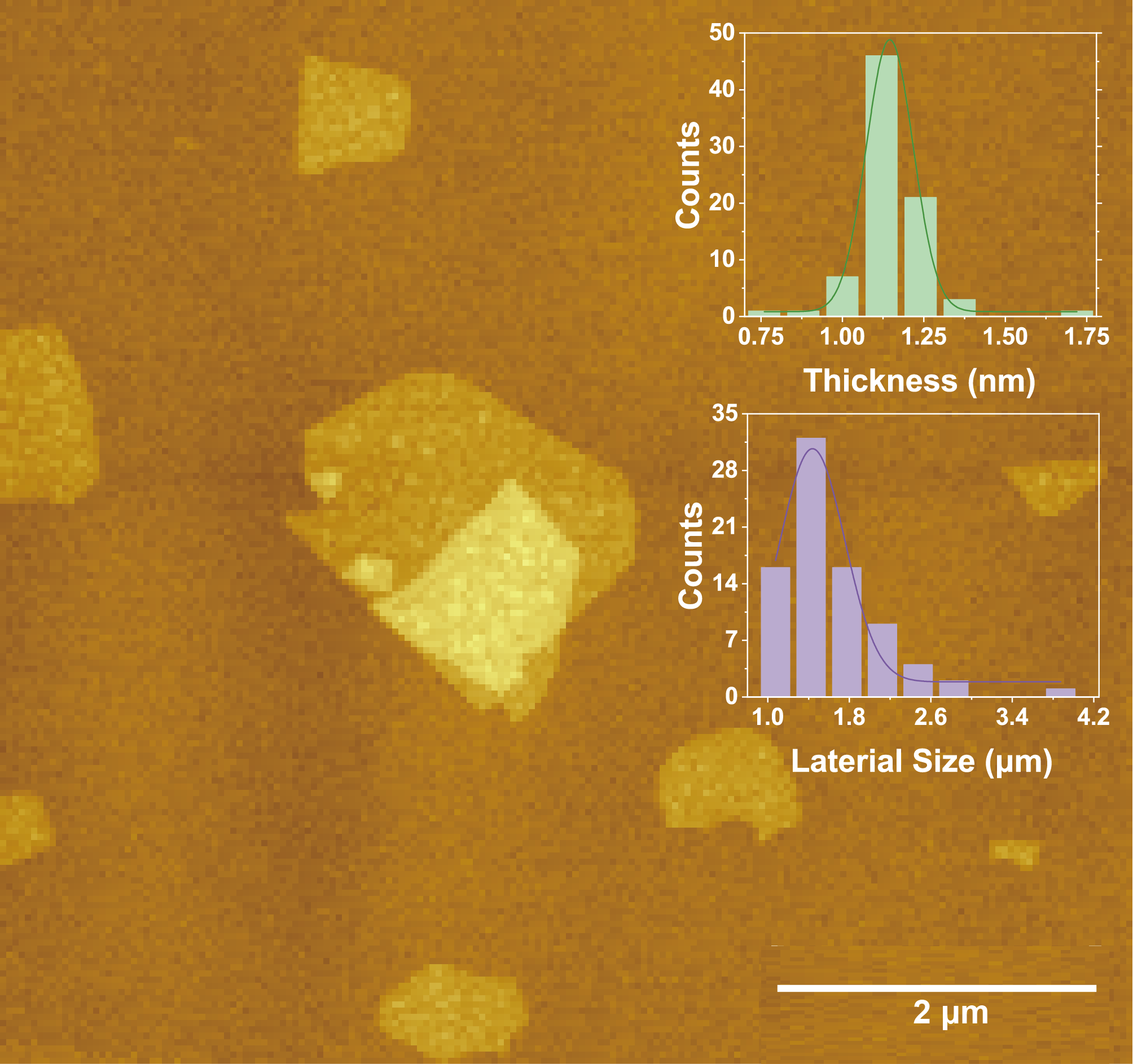


**Fig. S2** AFM image of 2D TiO2


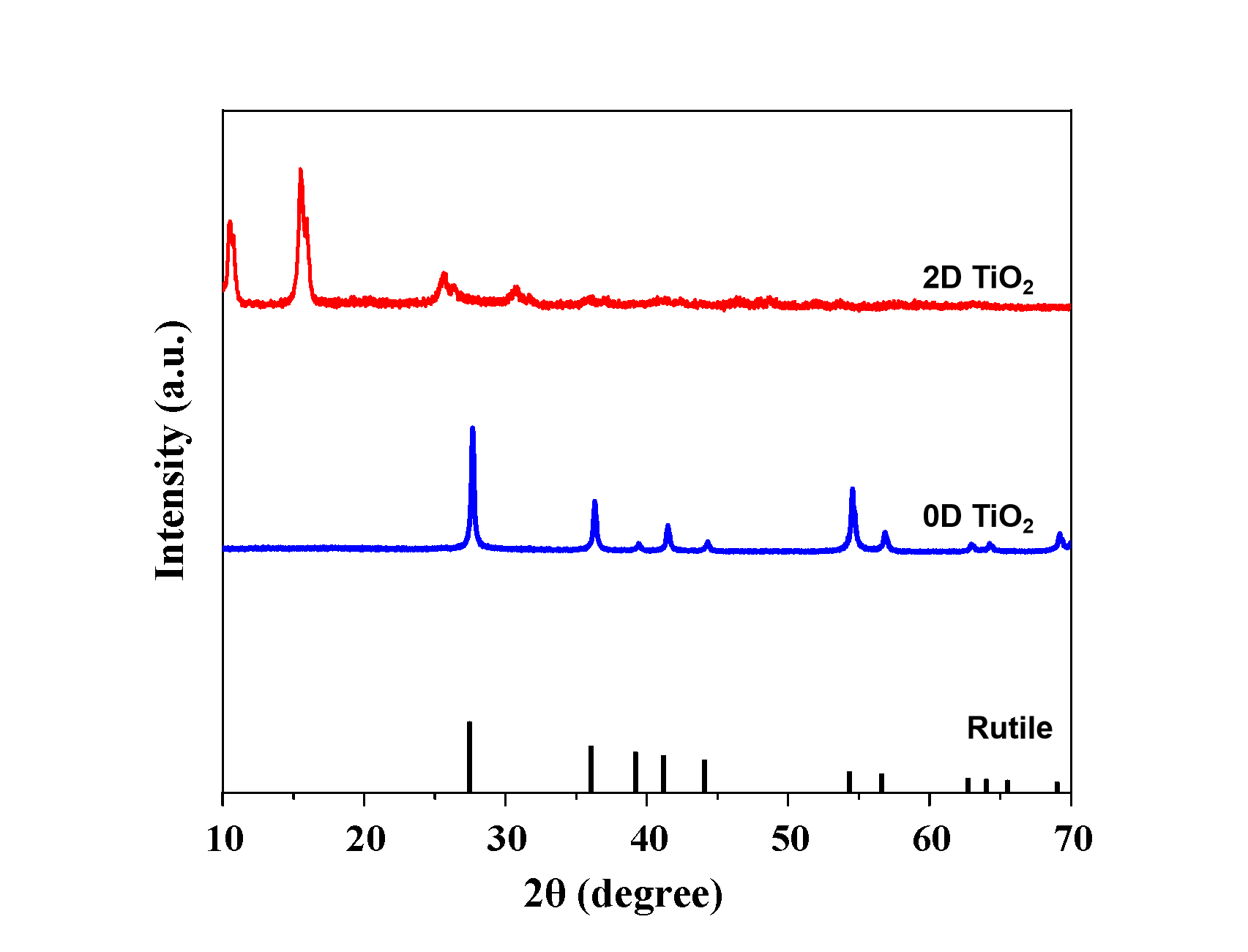


**Fig. S3** Comparative XRD analysis of 0D versus 2D TiO₂


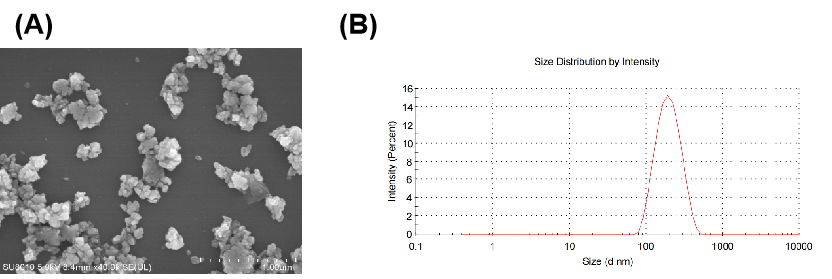


**Fig. S4** (**A**) SEM image of nanoscale TiO2 particles. (**B**) DLS particle size analysis indicating a size distribution of approximately 100-200 nm


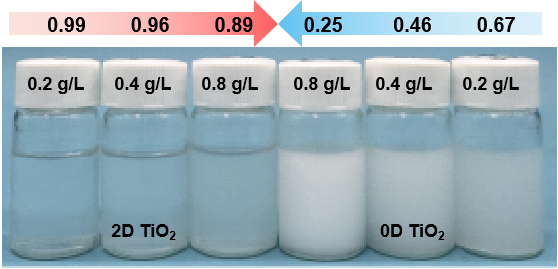


**Fig. S5** Photographs from side views of 0.2, 0.4, and 0.8 g/L aqueous 2D TiO2 solution (left to right) and 0D TiO2 solution (right to left), with the corresponding NAF marked in the arrow above


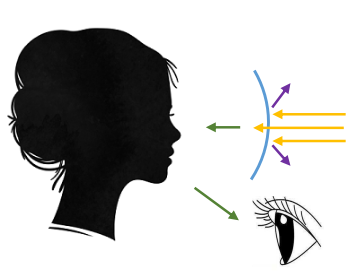


**Fig. S6** Natural Appearance Factor (NAF) Definition Diagram, where yellow arrows represent all incident light, purple arrows represent all blocked light, and green arrows represent light reflected from the skin surface to the human eye

**Fig. S7** Optical simulation of 2D TiO2, showing changes in (**A**) reflection, and (**B**) absorption with increasing thickness

**Table S1** Effects of different modification processes on the phototoxicity of TiO₂

| Materials system | Photoregulation mechanism | Phototoxicity | Application | References |
| --- | --- | --- | --- | --- |
| TiO2/Co3O4/Ti3C2T*x* | Bandgap narrowing was achieved through Co₃O₄ compositing and Ti3C2T*x* doping | Relatively high | Degradation of methylene blue | [S6] |
| Lignin@0D TiO2 | Coating with natural antioxidants to scavenge free radicals before they escape from TiO₂ particles | Relatively low | Sunscreen | [S7] |
| Benzophenones@TiO2 | Coated with organics but the coating gets damaged under UV irradiation | Relatively high | Sunscreen | [S8] |
| 1D TiO2 | Morphology control | Relatively high | Sunscreen | [S9] |
| 2D TiO2 | Morphology control | Almost none | Sunscreen | Our work |


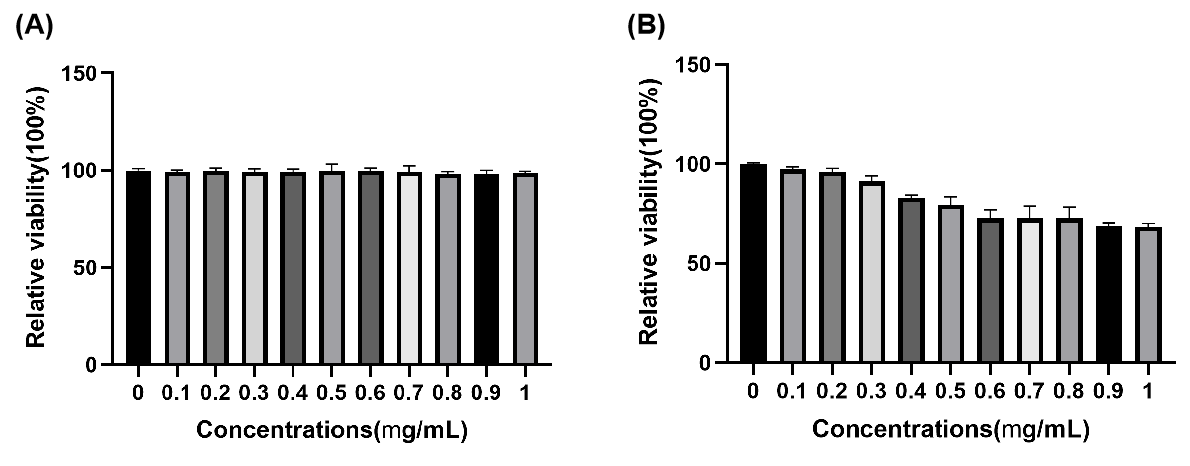


**Fig. S8** Cytotoxicity of TiO2 in co-culture with HeLa cells. (**A**) 2D TiO2, (**B**) 0D TiO2


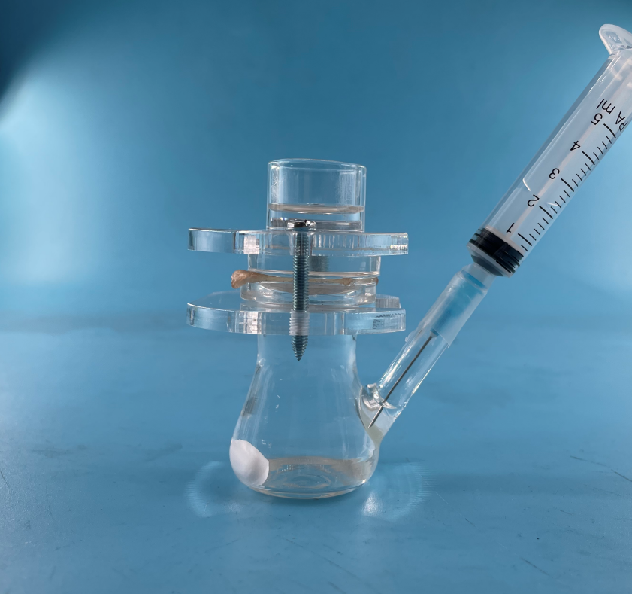


**Fig. S9** Franz diffusion cell for in vitro permeability testing


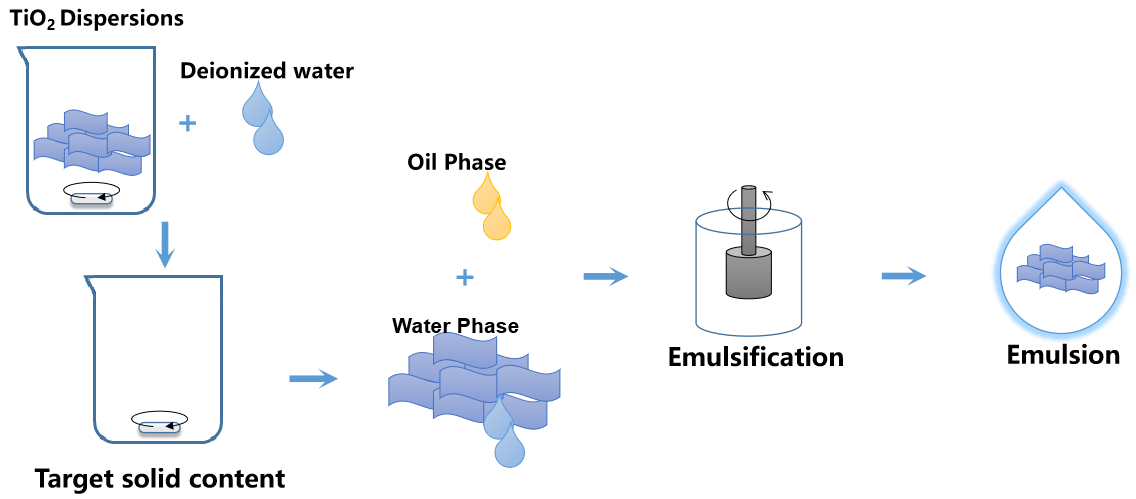


**Fig. S10** Synthesis process of the minimalist formula emulsion


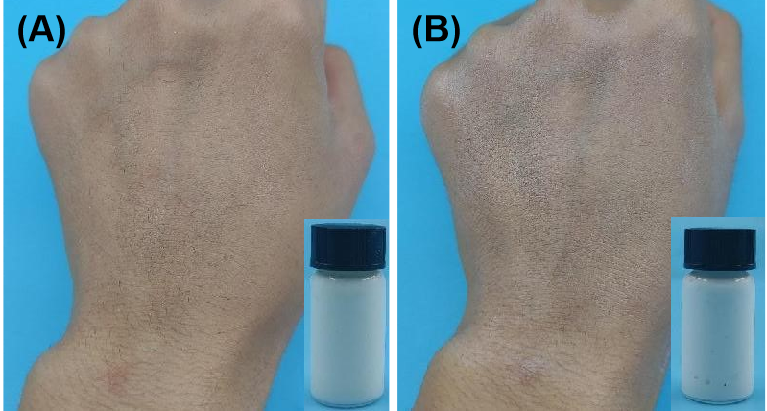


**Fig. S11** Appearance of the emulsion on the back of a human hand after application. Emulsion (**A**) micro-sheets, (**B**) nanoparticles


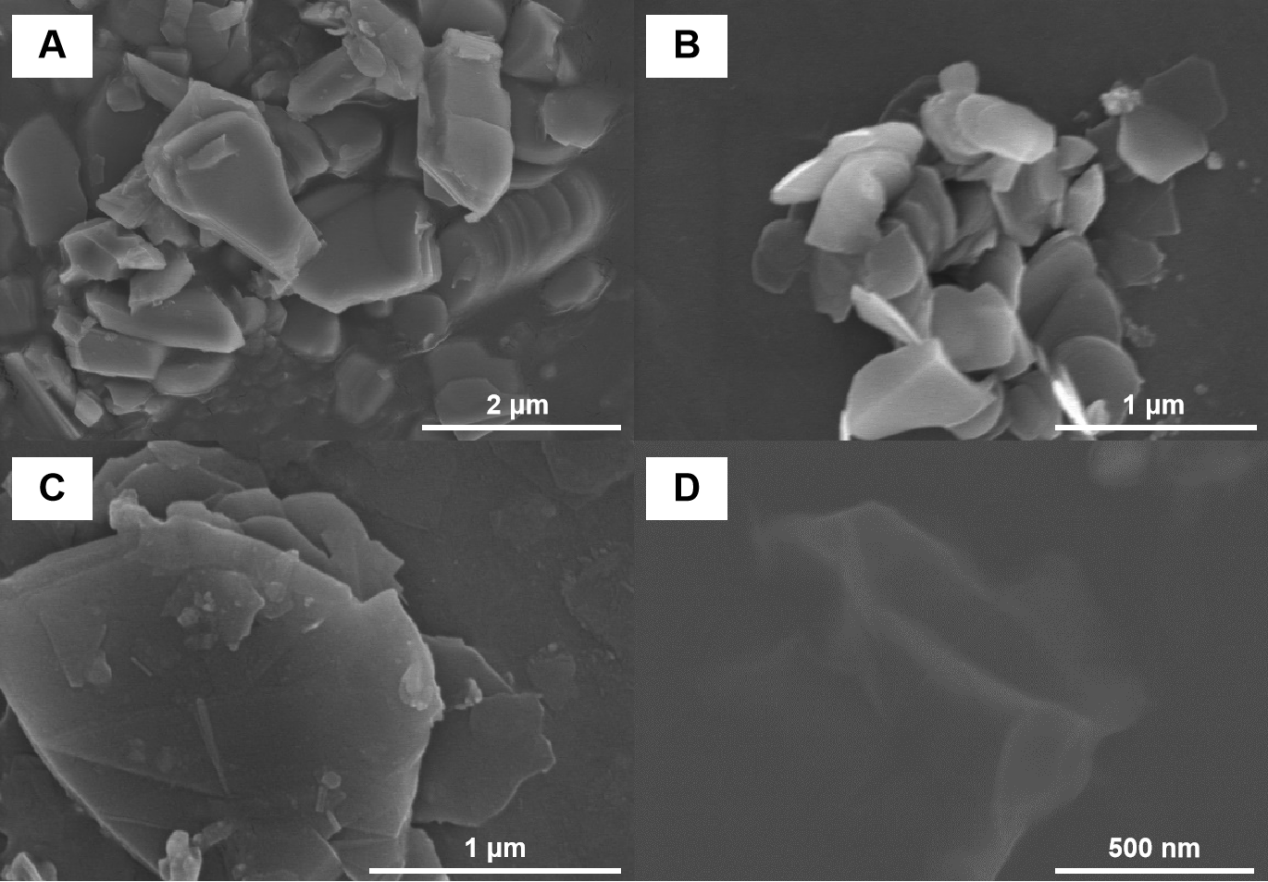


**Fig. S12** Synthesis process of 2D Fe-doped TiO2 . (**A**) Pre-sintered iron-doped KTLO. (**B**) Precursor after hydrogen ion intercalation. (**C**) Precursor with water molecule intercalation. (**D**) 2D Fe-doped TiO2


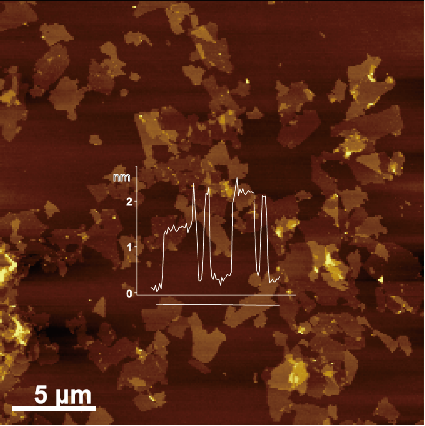


**Fig. S13** AFM image of 2D Fe-doped TiO2


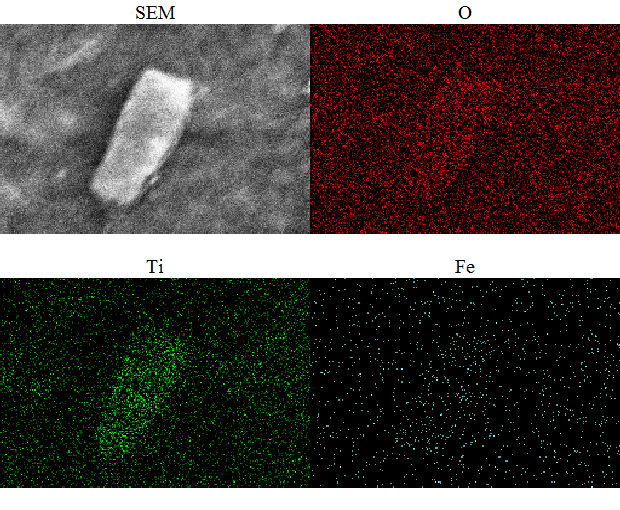


**Fig. S14** Elemental distribution of 2D Fe-doped TiO2


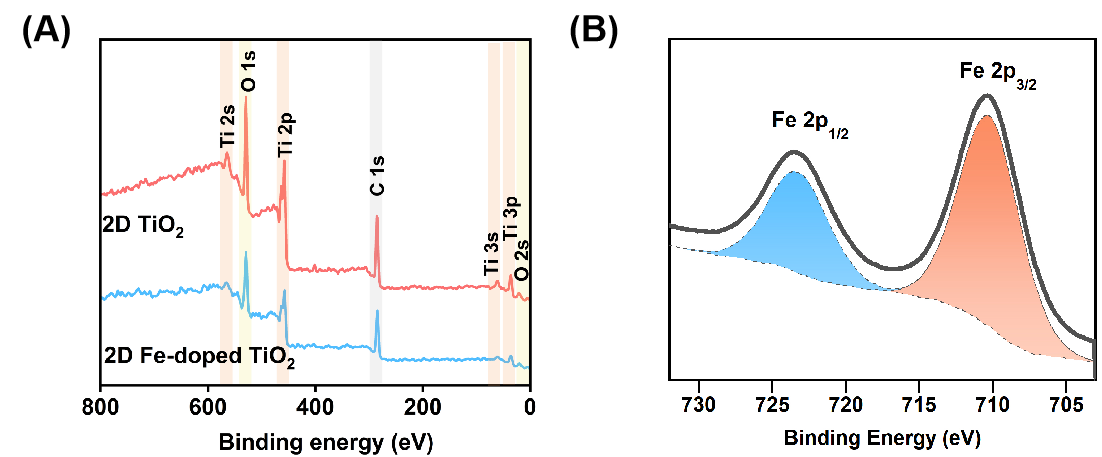


**Fig. S15** XPS analysis of TiO2: (**A**) XPS survey spectrum of 2D TiO2 and Fe-doped TiO2, (**B**) Fe 2p spectra


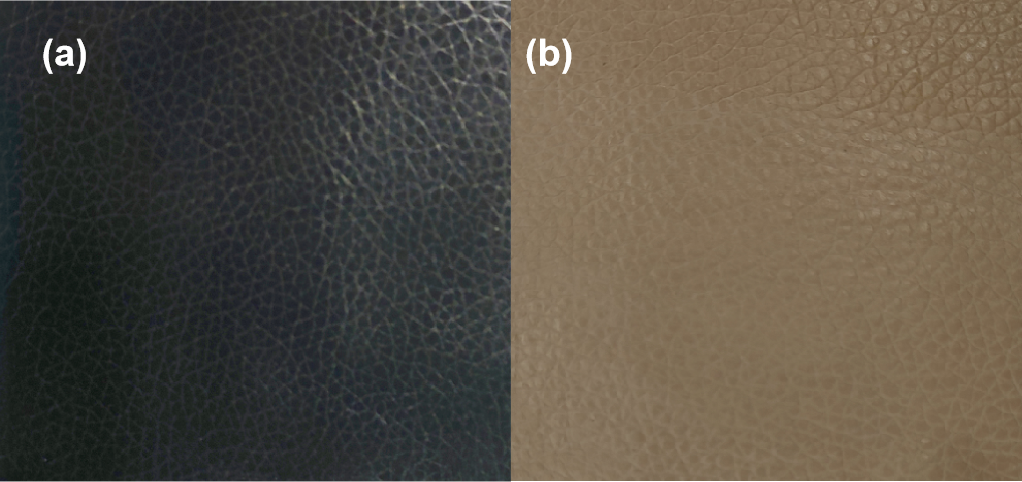


**Fig. S16** Image of an emulsion containing 4w/w% 2D Fe-doped TiO2 applied on synthetic leather


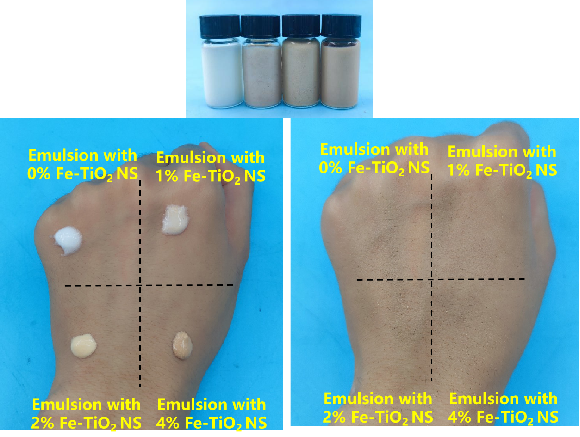


**Fig. S17** Appearance of the emulsion on the back of a human hand after application


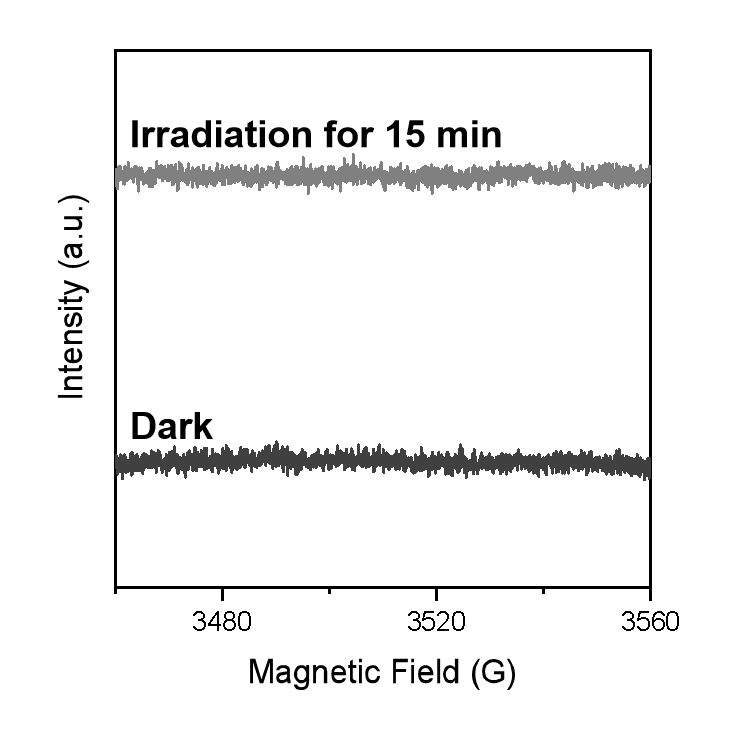


**Fig. S18** EPR spectrum of 2D Fe-doped TiO2 under UV irradiation showing no detected reactive oxygen species


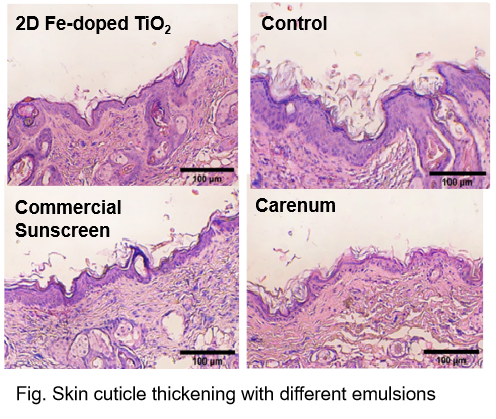


**Fig. S19** H&E stained histological sections of mouse skin epidermis, corresponding to sunscreens containing 2D Fe-doped TiO2, commercial sunscreen, emulsion base, and normal skin


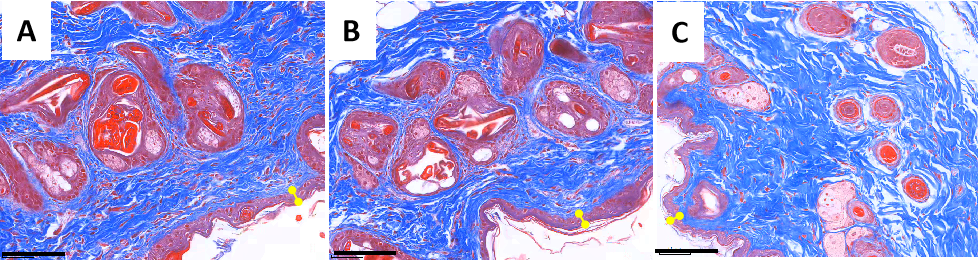


**Fig. S20** Masson stained histological sections of keratin in nude mouse skin, corresponding to the sunscreens containing 2D Fe-doped TiO2, commercial sunscreen, emulsion base. All scale bar, 100 μm

**Supplementary References**

1. T. Sasaki, M. Watanabe, H. Hashizume, H. Yamada, H. Nakazawa, Macromolecule-like aspects for a colloidal suspension of an exfoliated titanate. pairwise association of nanosheets and dynamic reassembling process initiated from it. J. Am. Chem. Soc. **118**(35), 8329–8335 (1996). <https://doi.org/10.1021/ja960073b>
2. G. Liu, L. Wang, C. Sun, X. Yan, X. Wang et al., Band-to-band visible-light photon excitation and photoactivity induced by homogeneous nitrogen doping in layered titanates. Chem. Mater. **21**(7), 1266–1274 (2009). <https://doi.org/10.1021/cm802986r>
3. G. Liu, L. Wang, C. Sun, Z. Chen, X. Yan et al., Nitrogen-doped titania nanosheets towards visible light response. Chem. Commun. (11), 1383–1385 (2009). <https://doi.org/10.1039/B820483G>
4. J. Chandezon, G. Raoult, D. Maystre, A new theoretical method for diffraction gratings and its numerical application. J. Opt. **11**(4), 235 (1980). <https://doi.org/10.1088/0150-536X/11/4/005>
5. M.G. Moharam, E.B. Grann, D.A. Pommet, T.K. Gaylor, Formulation for stable and efficient implementation of the rigorous coupled-wave analysis of binary gratings. J. Opt. Soc. Am. A **12**(5), 1068–1076 (1995). <https://doi.org/10.1364/JOSAA.12.001068>
6. R. Ghamarpoor, A. Fallah, T. Eghbali, Design of bifunctional sandwich-like Co@Si/Ox-MXene nanocomposite to increase the supercapacitor properties and removal of pollutants from wastewater. J. Alloys Compd. **983**, 173920 (2024). <https://doi.org/10.1016/j.jallcom.2024.173920>
7. M. Morsella, N. D’Alessandro, A.E. Lanterna, J.C. Scaiano, Improving the sunscreen properties of TiO2 through an understanding of its catalytic properties. ACS Omega **1**(3), 464–469 (2016). <https://doi.org/10.1021/acsomega.6b00177>
8. Q. Guo, D. Wei, C. Zhao, C. Wang, H. Ma et al., Physical UV-blocker TiO2 nanocomposites elevated toxicity of chemical sunscreen BP-1 under UV irradiation. Chem. Eng. J. **469**, 143899 (2023). <https://doi.org/10.1016/j.cej.2023.143899>

[S9] S. Limsakul, T. Mahatnirunkul, C. Phromma, T. Chomtong, N. Cholnakasem et al., Novel physical sunscreen from one-dimensional TiO2 nanowire: synthesis, characterization and the effects of morphologies and particle size for use as a physical sunscreen. Nano Struct. Nano Objects **35**, 101027 (2023). <https://doi.org/10.1016/j.nanoso.2023.101027>
